# Supplementary material for: A Comprehensive Review of Hypotheses About the Biological Function of Zearalenone, and a New Hypothesis for the Function of Resorcylic and Dihydroxyphenylacetic Macrolactones in Fungi
Source: Toxins (Basel). 2025 May 3;17(5):226. doi: 10.3390/toxins17050226 (PMC12115441; doi:10.3390/toxins17050226)
Supplement: Supplementary file 1 [file toxins-17-00226-s001.zip › toxins-3590747-supplementary.pdf]

Supplementary Materials: A Comprehensive Review of Hypotheses About the Biological Function of Zearalenone, and a New Hypothesis for the Function of Resorcylic and Dihydroxyphenylacetic Macrolactones in Fungi

María Viñas and Petr Karlovsky

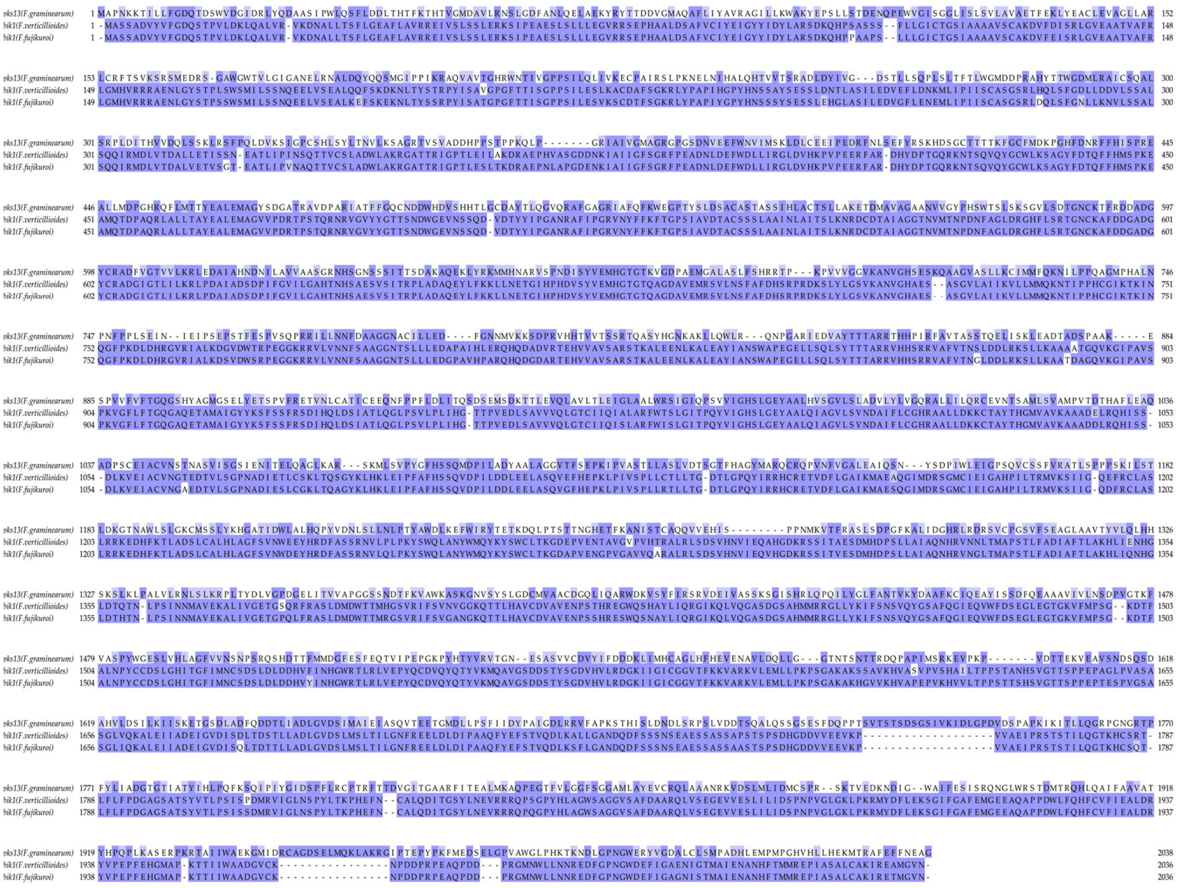

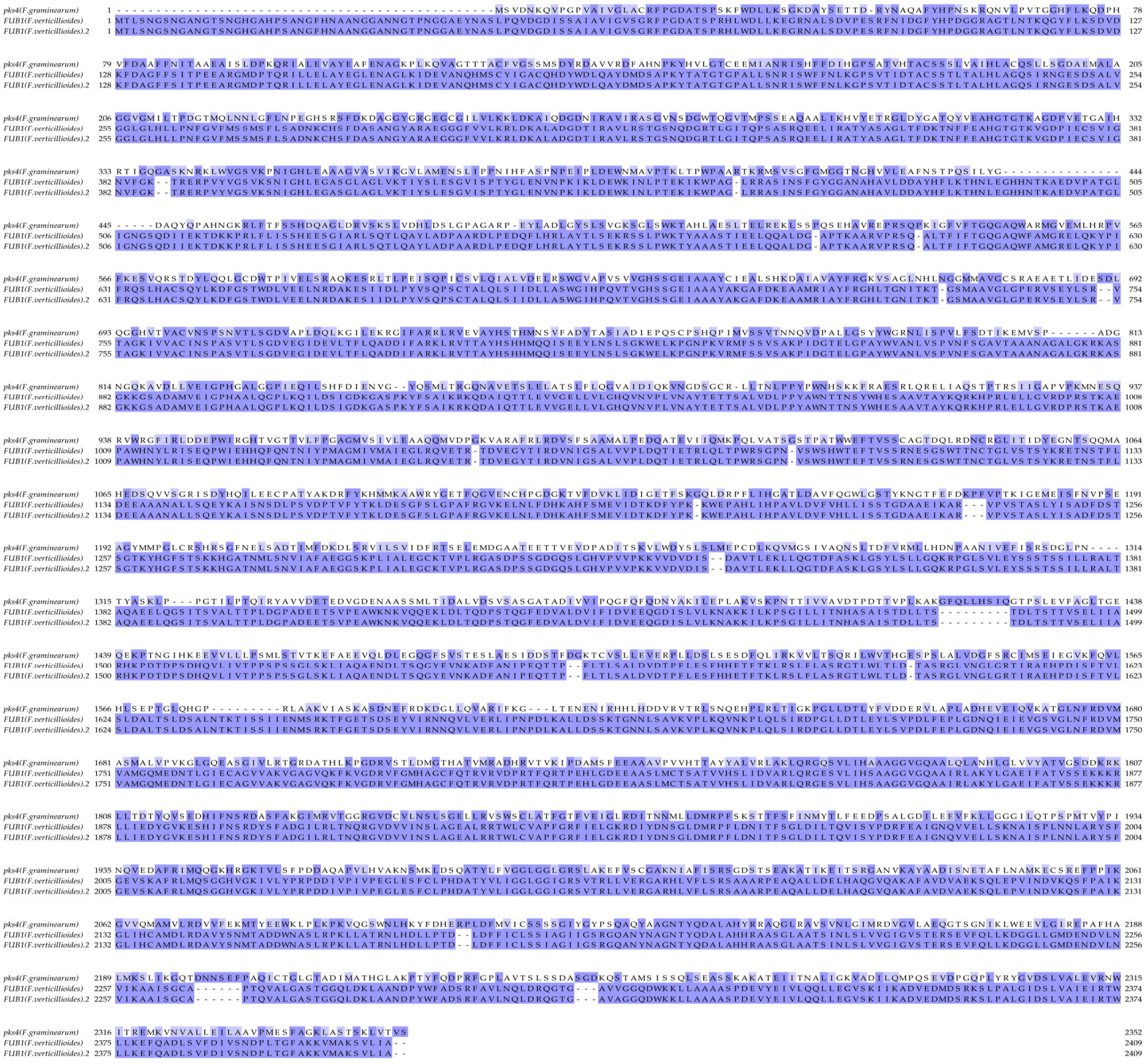

Figure S1. Alignment of PKS4 and PKS13 from *F. graminearum* with protein sequences from *F. verticillioides* that have been claimed to be involved in ZEN synthesis, but in reality are involved in the synthesis of bikaverin and fumonisin.

Table S1. Putative assignments of PKS4- and PKS13-homologues to known RALs.

Species name	PKS13-like proteins		PKS4-like proteins		Product
	Primary accession (UniProt)	Identity (%)	Primary accession (UniProt)	Identity (%)	Name
Lasiodiplodia hormozganensis	A0AA39XQ95	59.28	A0AA39XQ93	61.51	Hypothemycin
Lasiodiplodia theobromae	X4Y4S0	57.55	A0A5N5D0W3	62.90	Hypothemycin
Niveomyces insectorum	A0A167TEN1	57.53	A0A167TES2	63.19	Hypothemycin
Staphylotrichum longicolle	A0AAD4I0I1	57.49	A0AAD4EYD6	59.34	Radicicol
Colletotrichum sublineola	A0A066XKJ9	54.79	A0A066XQB6	58.78	Radicicol
Talaromyces proteolyticus	A0AAD4L1W9	53.58	A0AAD4L7J8	59.10	Radicicol
Colletotrichum fioriniae	A0A010R167	53.31	A0A010RCC8	62.88	Cladosporin
Colletotrichum sojae	A0A8H6JIU0	53.16	A0A8H6JIV5	63.01	Cladosporin
Colletotrichum musicola	A0A8H6J5Q8	52.96	A0A8H6KDV5	63.09	Cladosporin
Colletotrichum costaricense	A0AAI9YJ29	52.70	A0AAI9YJ28	62.82	Cladosporin
Colletotrichum navitas	A0AAD8PP42	51.41	A0AAD8PNH4	56.95	Radicicol
